# Effects of human chorionic gonadotropin intrauterine injection on oocyte retrieval day on assisted reproductive techniques outcomes: An RCT

**DOI:** 10.18502/ijrm.v19i9.9709

**Published:** 2021-10-10

**Authors:** Robabe Hosseinisadat, Lida Saeed, Sareh Ashourzadeh, Sedigheh Safar Heidari, Victoria Habibzadeh

**Affiliations:** ^1^Department of Obstetrics and Gynecology, School of Medicine, Kerman University of Medical Sciences, Kerman, Iran.; ^2^Kerman Infertility Center, Afzalipour Hospital, Kerman University of Medical Sciences, Kerman, Iran.

**Keywords:** Oocyte retrieval, Chorionic gonadotropin, Pregnancy, Assisted reproductive techniques.

## Abstract

**Background:**

Several mediators play an important role in implantation. One of these mediators is human chorionic gonadotropin (HCG).

**Objective:**

To evaluate the effects of HCG intrauterine injection on the day of oocyte retrieval on the result of assisted reproductive techniques (ART).

**Materials and Methods:**

In this randomized clinical trial study, 126 women who were referred to Afzalipour Infertility Center between December 2018 to December 2019 undergoing in vitro fertilization/intracytoplasmic sperm injection cycles were enrolled and assigned to two groups of: a case (n = 62) and a control group (n = 64). The protocols for both groups were the same; except that the case group was injected with the protocols for both groups were the same, except that the case group was injected with 1000 IU of HCG into uterine cavity following the oocyte puncture, while no medication was administered to the control group. The implantation rate, chemical pregnancy, clinical pregnancy, and abortion rates were compared between the two groups.

**Results:**

Positive chemical pregnancy was seen in 15 (27.3%) cases of the case group and 14 (25.5%) of the control group. No significant difference was seen in the chemical and clinical pregnancy rates between the groups. The abortion rate was higher in the control group but that was not significant.

**Conclusion:**

A 1000 IU of HCG intrauterine injection after oocyte retrieval does not improve implantation, chemical or clinical pregnancy rates in ART cycles. Further studies are needed to clearly understand the role of HCG intrauterine injection in the day of oocyte retrieval in ART outcomes.

## 1. Introduction

Successful pregnancy depends on successful implantation (1). In assisted reproductive techniques (ART) cycles, several factors such as embryo quality and endometrial receptivity can affect successful implantation (2, 3). It is estimated that implantation failure is responsible for about 50-75% of abortions and pregnancy losses (4, 5). Implantation is a complex process and several factors regulate this process. When implantation is in progress, in the first step, the embryo attaches to the maternal endometrium and starts embryo-maternal interchange (1, 6). In maternal side, steroid hormones such as estrogens and progesterone play an important role in endometrial receptivity (7). At the fetal level, several mediators play an important role; one of them is human chorionic gonadotropin (HCG) (8).

HCG is the main factor in various stages of pregnancy progression such as existence of corpus luteum, motivation of progesterone secretion, support of fetal implantation, regulator of the trophoblast cells to distinction, angiogenesis inspiration, and finally embryo-maternal adjustment (9, 10). Recently, much attention has been paid to the HCG role in preparing the uterine cavity condition for implantation (11, 12).

HCG inhibits some markers of decidualization such as macrophage colony-stimulating factor and insulin-like growth factor-binding protein 1 (13). HCG can stimulate leukemia inhibitory factor (cytokine required for implantation), matrix metalloproteinase-9 (tissue remodeling regulator), and vascular endothelial growth factor (an angiogenic growth factor) (12, 14). These changes in the uterus are the paracrine effects of HCG on cells remodeling, implantation, vascularization, and angiogenesis, which can increase the likelihood of successful implantation.

It has been reported that a 500 IU HCG injection into the uterine cavity on the embryo transfer (ET) day can significantly increase the implantation and chemical, clinical, and ongoing pregnancy rates and also the live delivery rate (15). In another study, researchers reported that there was no significant difference between the implantation or pregnancy rates in groups given a 500 or 1000 IU HCG intrauterine injection before ET and a control group (16).

Several dosages of HCG on the ET day have been evaluated by previous studies, however, to the best of our knowledge, none of them studied the effect of a 1000 IU HCG injection into the uterine cavity immediately after oocyte retrieval. Thus, the aim of this study was to evaluate the effects of intrauterine HCG injection on the oocyte retrieval day on ART outcomes.

## 2. Materials and Methods

In this randomized, prospective, unblended, clinical trial, 126 women who were referred to the Afzalipour Infertility Center and underwent in vitro fertilization (IVF)/intra cytoplasmic sperm injection (ICSI) cycles from December 2018 to December 2019 were enrolled. Participants were randomly divided into two groups of case (n = 62) and control (n = 64) group using random number table.

The inclusion criteria were infertile women aged < 40 yrs. Women aged > 40 yr, having Azoospermic partners, suffering from uterine leiomyoma with endometrial pressure, or endometriosis, history of recurrent implantation failure, those who had failed to achieve a clinical pregnancy after transfer at least three good-quality embryo transfer, endocrine disease, and hydrosalpinx were excluded.

The sample size of the study was calculated based on the previous study (17). The clinical pregnancy rates were 59.2% and 31.3% in the case and control groups, respectively. Considering these rates, and with α = 0.05, a sample size of 55 women in each group was calculated.

All women in the IVF/ICSI cycle received routine treatment. All women were on a short and flexible antagonist protocol for controlled ovarian stimulation. Gonadotropin Cinal F (Cinal F, CinnaGen, Iran) or HMG (PDHOMOG, Pooyesh Darou, Iran) or the combination of the two was administered from day two of menstruation. The initial dose of gonadotropin (150-450 IU/day) was prescribed based on women age and weight. On the sixth day monitoring was started. In continue when the dominant follicle size reached 12 mm Antagonist (Cetrorelix, Merck-Serono, Germany) was prescribed. Finally transvaginal ultrasonography was done and when at least two follicles were seen that sizes were upper than 18 mm, triggering was complete by an intramuscular injection of 10,000 IU hCG (PDPREG, Pooyesh Darou, Iran). After 36-40 hr, oocyte puncture was performed with vaginal ultrasound and general anesthesia.

In the case group, after the oocyte puncture, 5000 units of HCG (Pooyesh Darou PDP PREG, Iran) were dissolved in 5 cc of normal saline. 1 cc of this solution, which was equivalent to 1000 units, injected into the uterus, with an intrauterine insemination catheter (Sperm Trans, India). In the control group, the protocol was the same as the case group, except no medication was injected into the uterine cavity on the day of oocyte retrieval.

All oocytes were fertilized by IVF/ICSI and the embryos were transferred three days after the oocyte retrieval. The implantation rate (5 wk after embryo transfer) was measured based on the number of gestational sacs seen on sonography relative to the number of transferred embryos. Chemical and clinical pregnancies were determined by measuring βHCG two wk after the ET and by observing the fetal heart rate two-three wk after the positive pregnancy test through ultrasound, respectively. The abortion rate was defined as pregnancy losses before the 20${}^{\mathrm{th}}$ wk of gestation per chemical positive pregnancy.

### Ethical considerations

This study was approved by the ethics committee of Kerman University of Medical Sciences, Kerman, Iran (Code: IR.KMU.REC.1397.070) and is registered with the Iranian Registry of Clinical Trials (IRCT) under code IRCT20151004024335N3. Also, a written informed consent was obtained from all cases prior to the study.

### Statistical analysis

The Statistical Package for the Social Sciences software version 20 (SPSS, IBM Co., Illinois, USA) was used for the statistical analysis. Student's *t* test and proportional test were performed for the numerical variables and categorical variables, respectively. The results were presented as mean ± SD or as a frequency percentage (%). A p-value < 0.05 was considered statistically significant.

## 3. Results

**Figure 1:**
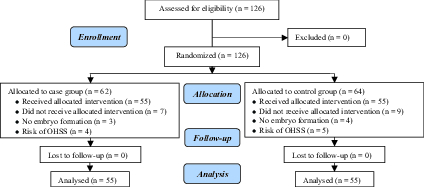
Consort flow diagram of the study.

Out of the 126 women participating in the study, seven in the case group (three cases without embryo formation and four cases at risk of ovarian hyperstimulation syndrome and nine in the control group (four cases without embryo formation and 5 cases at ovarian hyperstimulation syndrome risk) were excluded from the study. Finally, 55 cases in each group were studied and analyzed (Figure 1).

The basic and demographic characteristics of the participants in the two study groups were examined. There was no statistically significant difference in the mean age, infertility duration, type, or etiology of infertility between the two groups (Table I).

The ART primary outcomes were similar in groups. There was no statistically significant difference between the two groups in terms of oocyte number, matured oocyte (M2), fertilized oocytes (2PN), or mean number of transferred embryos (Table II).

Table III presents the secondary outcome of ART in the HCG group compared to the control group. The implantation rate was similar between the two groups (15 gestational sacs in each group). Chemical pregnancy was positive in 15 cases of the HCG group and in 14 cases of the control group. There was no statistically significant difference in the implantation rate or chemical pregnancy rate between the two groups (p = 0.9). Clinical pregnancy was positive in 13 cases of each group. No statistically significant difference was seen in the chemical or clinical pregnancies between the two groups. Although the abortion rate in the control group was higher than the HCG group (42.9% vs 26.7%), the difference was not statistically significant (Table III).

**Table 1:** Basic and demographic characteristics of participants in the two study groups


		**HCG group**	**Control group**	**p-value**
Age*		30.41 ± 4.78	31.37 ± 6.63	0.44
Infertility duration*		5.37 ± 2.88	6.57 ± 4.13	0.08
BMI*		26.07 ± 4.23	26.10 ± 4.23	0.96
Endometrial thickness*		9.24 ± 1.86	9.60 ± 1.62	0.28
Infertility type**				
	**Primary**	40 (72.7)	39 (70.9)	
	**Secondary**	15 (27.3	16 (29.1)	0.83
Infertility etiology**		14 (25.5)	17 (30.9)		
**Ovarian factor**		3 (5.5)	2 (3.6)	
**Tubal factor**		19 (34.5)	15 (27.3)	
**Male factor**		2 (3.6)	4 (7.3)	
**Mixed unknown**		17 (30.9)	17 (30.9)	0.80
*Data presented as Mean ± SD, **n (%). Student's *t* test. HCG: Human chorionic gonadotropin, BMI: Body mass index				

**Table 2:** Primary outcome of ART in the HCG group compared to the control group


	**HCG group**	**Control group**	**p-value**
**Oocyte number**	12.74 ± 5.55	10.69 ± 5.46	0.053
**M2**	10.83 ± 5.36	9.67 ± 5.05	0.244
**2PN**	6.60 ± 4.35	5.76 ± 3.29	0.258
**Embryo number**	4.90 ± 3.59	4.32 ± 2.75	0.343
**Transferred embryo**	1.98 ± 0.45	2.09 ± 0.55	0.260
Data presented as Mean ± SD. Student's *t* test. HCG: Human chorionic gonadotropin, M2: Matured oocyte, 2PN: 2 pronucleus			

**Table 3:** Secondary outcome of ART in the HCG group compared to the control group


	**HCG group**	**Control group**	**p-value**
**Implantation rate (sac/transferred embryo)**	15/109 (13.76)	15/115 (13.04)	0.963
**Chemical pregnancy (positive βHCG)**	15 (27.3)	14 (25.5)	0.829
**Clinical pregnancy (gestational sac)**	13 (23.6)	13 (23.6)	1.000
**Abortion rate**	4 (26.7)	6 (42.9)	0.359
Data presented as n (%). Student's *t* test. HCG: Human chorionic gonadotropin			

## 4. Discussion 

Our results showed that positive chemical pregnancy occurred in 15 (27.3%) cases of the case group and 14 (25.5%) of the control group. No significant difference was seen in the chemical or clinical pregnancy rates between the groups. The abortion rate was higher in the control group but not significant.

The implantation of an embryo in the uterus is a complex process that involves many molecular processes (1). Implantation and the interaction between the embryo and endometrium in ART cycles depend on many factors including endometrial receptivity, embryo quality, and, importantly, HCG (2). HCG, as a luteinizing hormone (LH) homologous isomer has a common receptor with LH, which is named LHCGR. So a combination of LH and HCG can regulate embryo implantation in the endometrium (18, 19).

HCG plays an important role in regulating cytokine secretion from the proliferative to the secretory phase of the endometrium and especially at the time of implantation. Consequently, HCG may show a complementary character in embryo implantation by regulating molecular signaling pathways (20).

Several scientific researchers have investigated the positive effect of HCG on the endometrium and mutual linking between the embryo and endometrium for progress in the implantation process (12, 21). Many studies have shown that HCG is the primary hormone secreted by a newly formed embryo in the uterine cavity to promote other molecular signaling pathways, in order to protect endometrial thickness and support the implantation (22, 23).

Studies have reported beneficial effects of HCG injection before ET on the endometrium and thereby on the HCG created by the embryo before its implantation (16, 24). Therefore, the current study aimed to evaluate the effect of intrauterine injection of HCG into the uterine cavity on the day of oocyte retrieval. We hypothesized that HCG may need more time to show its positive effects on the endometrium. To date, to the best of our knowledge, only one study has investigated the beneficial effects of intrauterine injection of HCG immediately after oocyte retrieval (16).

Our results showed that an intrauterine HCG injection on the oocyte retrieval day did not have any effects on the pregnancy rate. All of our cases had similar ovulation induction and received fresh embryo transfer. Our results showed that an intrauterine injection of HCG on the oocyte retrieval day did not improve implantation and/or the chemical pregnancy rate compared with the control group.

In a study published in 2011, injections of 100, 200, and 500 IU of HCG seven min before the ET were compared with each other and with a control group. The injection of 500 IU of HCG caused a significant increase in the pregnancy rate, but there was no change in the pregnancy rate in the groups of 100 and 200 IU. In their study, the injection was performed on the day of transfer and shortly before the transfer of the embryo into the endometrial cavity. Also, in this study, HCG was diluted using an embryo culture medium, however, in our study, normal saline was used. In the present study, the dose of the HCG was 1000 IU, which was higher than in the mentioned study. The transfer time, injection dosage; medicine concentration, and preparation of HCG could have been responsible for the insignificant results of our study compared to the mentioned study (17).

In the Aaleyasin study, on the day of transfer, 500 IU of HCG dissolved in 0.05 cc of culture medium and injected into the uterine cavity using a transfer catheter. The embryos were injected into the uterus five-seven min later using another transfer catheter. In our study, the HCG was injected at a higher dose using an IUI catheter. The difference in the catheter could be the reason for the increased rate of pregnancy in Aaleyasin's paper, however, due to the restrictions on the import of catheters in Iran, the transfer catheter was unavailable to us. The use of this catheter is suitable for special cases such as repeated implantation failure (RIF) (15).

In 2016, researchers injected 500 and 1000 IU of HCG diluted in a culture medium into the uterine cavity, seven min before the ET using a transfer catheter. In another group, nothing was injected into the uterus. The pregnancy rate and the IVF outcomes were similar in all three groups. In their study, as in ours, women with endometriosis and RIF were excluded, however, the difference in the results might have occurred due to the exclusion of risky subgroups with less ART success (16).

In a study, similarly to ours, the injection was performed on the oocyte retrieval day using an IUI catheter, but unlike in our study, it caused a significant increase in pregnancy and implantation. In our study, 1000 units of HCG in 1 cc of normal saline were injected, but in the Navali study, 500 units were injected in 0.5 cc of normal saline. They injected normal saline as a placebo in the control group. The difference in the results may be due to the differences in the dose and volume of the drug and placebo injections. In their study, women with low ovarian reserve were excluded, which was not an exclusion criterion of our study. HCG injections may work better in people with higher ovarian reserve. Given that studies similar to ours and Navali's are rare, further studies with different doses, volumes, and dilutions are recommended (24).

In a paper published in 2014, 500 IU HCG was diluted in a culture medium and injected into the uterine cavity using a transfer catheter, three min before the ET. They also transferred embryos in blastocyst stage using another transfer catheter. In their study, as in ours, the pregnancy rate did not increase. So, they concluded that an injection of 500 IU HCG on the day of transfer does not improve pregnancy in blast ET (25).

Researchers in 2019 injected recombinant HCG into the uterine cavity in women with endometriosis before frozen-thawed embryo transfer (FET). The injection was given one day before the FET using an IUI catheter. In these women, the rates of pregnancy and live birth were significantly higher than in the control group. In our study, women with endometriosis were excluded. HCG injections may be more effective in groups of cases with other infertility problems. Studies can be done on FET cycles as well as on different types of infertility (26).

Recently in a study injected 500 IU of HCG in 0.05 cc of culture medium into the uterine cavity using an IUI catheter, three days before the ET in RIF cases and in the FET cycles. Their results showed an increase in the pregnancy rate. The volume and dosage of the HCG were lower in their study than in ours. Also, in our study, women with the RIF were excluded. HCG injections may be more effective in women with a lower chance of IVF success than in normal cases (27).

In the present study, IU HCG injection was performed on the oocyte retrieval day and the patient was in complete anesthesia; therefore, the catheter was easily inserted into the uterine cavity and was painless reducing the patient's stress. We suggest that if the HCG injection on the oocyte retrieval day improves ART outcomes, it may be more useful than injection on the ET day, when the patient is not uner anesthesia and the injection may cause pain and stress.

## 5. Conclusion

We concluded that an intrauterine injection of 1000 IU of HCG after the oocyte retrieval does not improve implantation, chemical or clinical pregnancy rates in ART cycles. In this regard, more studies are needed to further examine clear the role of HCG intrauterine injection on oocyte retrieval day on ART outcomes. We suggest the researchers assess the effects of different dosages of HCG intrauterine injection, and the effects in women with different causes of infertility and in groups with a lower chance of ART success, such as women with RIF or endometriosis, etc. Also, the effects on ART outcomes of a larger sample size and injecting at the time of oocyte retrieval compared with on the ET day need to be studied. In addition, studies comparing the intrauterine HCG injections in fresh and frozen-thawed ET cycles should also be conducted.

##  Conflict of Interest

The authors have no financial or nonfinancial conflict of interest.
